# Can Match-Mimicking Intermittent Practice Be Used as a Simulatory Training Mode of Competition Using Olympic Time Frame in Elite Taekwondo Athletes?

**DOI:** 10.3389/fphys.2019.00244

**Published:** 2019-03-20

**Authors:** Sheng-Ju Chuang, Yu-Chi Sung, Chung-Yu Chen, Yi-Hung Liao, Chun-Chung Chou

**Affiliations:** ^1^ de Duve Institute, Université Catholique de Louvain, Brussels, Belgium; ^2^ Department of Chinese Martial Arts, Chinese Culture University, Taipei City, Taiwan; ^3^ Department of Exercise and Health Science, University of Taipei, Taipei City, Taiwan; ^4^ Department of Exercise and Health Science, National Taipei University of Nursing and Health Sciences, Taipei City, Taiwan; ^5^ Physical Education Office, National Taipei University of Technology, Taipei City, Taiwan

**Keywords:** high-intensity interval exercise, heart rate, muscle damage, inflammation, full-contact competition

## Abstract

**Aim:** The physiological realities between Taekwondo (TKD) simulation kicking training and TKD competition according to the Olympic time frame remain unclear. The purpose of this study is to establish an Olympic match-simulated kicking model and compare its effects with real TKD competition on physiological challenges and hormonal responses during serial matches in elite athletes.

**Method:** Sixteen elite TKD athletes randomly were assigned into either a TKD match-simulated kicking group (TMSK; *N* = 8, age: 21.3 ± 0.2 years) or a simulated TKD competition group (STC; *N* = 8, age: 21.6 ± 0.5 years). Both groups performed either simulated kicking or TKD competitions in the same time-course order, and all physiological parameters and blood sampling time-points were identical between groups. The heart rate (HR) and rating of perceived exertion (RPE) were recorded during each match-simulated kicking and TKD competition session. Blood samples were obtained before competition (Pre-Comp.), after competition—in ths case meaning four consecutive matches (End-Comp.), and 24 h after the first match (Next day) for determination of biomarkers of muscle damage (myoglobin and CK), hematological profiles, and hormonal profiles (testosterone and cortisol).

**Results:** The responses of HR, RPE, and blood lactate levels during the consecutive testing sessions showed no differences between TMSK and STC. The changes in CK and myoglobin were greater in STC (*p* < 0.05), and a greater decrease in red blood cell (RBC) loss was observed in the STC group (*p* < 0.05). Compared with TMSK, the inflammatory state, reflected by the ratios of neutrophils-to-lymphocyte (NLR) and platelets-to-lymphocyte (PLR), was higher in STC (*p* < 0.05). Moreover, the catabolic state (cortisol/testosterone) was greater in STC than in TMSK (*p* < 0.05).

**Conclusion:** We demonstrated that, compared with TMSK, the STC produced greater muscle damage, inflammatory responses, and catabolic stress in the Olympic competition time frame in elite male TKD athletes. Although TMSK is capable of eliciting similar physiological challenges as TKD competition, the muscle damage and hormonal profiles provoked by TMSK were not comparable to TKD competition. Our findings provide science-based data and better understanding for coaches, athletes, and sports scientists to develop TKD-specific training programs for Olympic preparation.

## Introduction

Among competitive sports, combat/martial art sports are unique and contain several specific features, including short periods with extremely high intensity, intermittent exercise patterns, and frequent physical contact/impacts during competition events. Taekwondo (TKD), a traditional Korean martial art combat sport, has evolved into a modern Olympic discipline (Bridge et al., 2009). TKD is an intensive sport with a high demand for anaerobic energy (~30% of total metabolic work during combat) and is accompanied by tremendous muscle injury or soreness from intense eccentric contractions (Bridge et al., 2009; Crisafulli et al., 2009; Campos et al., 2012; Lopes-Silva et al., 2018) during athletic practice and competitions (Balnave and Thompson, 1993; Kwok, 2012). Furthermore, according to the Olympics and World Taekwondo (WT) regulations, TKD combats are comprised of at least three rounds of 2 min combat and 1 min break between each round (World Taekwondo, 2018), and the athletes may have to fight in consecutive schedules for at least 4–5 matches within a single day to be qualified to compete for the final championship. Consequently, the accumulative physiological fatigue during competitions is extreme in TKD athletes.

For coaches and athletes in combat sports, integrating tasks that represent formal competitive conditions into a regular training program is a common way to promote the transfer of combat skills from training to competition. To enhance practice, TKD athletes need to complete representative learning tasks for simulating key aspects of competition (Araújo et al., 2007). Accordingly, athletes not only have to perform TKD-specific skills and conditioning training but also have to participate in regional tournaments. However, these reality simulated training regimens (team combat practice, friendly match/competition, and regional tournament) possibly cause varying levels of sport injuries due to the frequent physical contact between athletes (Kazemi et al., 2005; Covarrubias et al., 2015; Hammami et al., 2018), because injuries are an inherent risk due to the nature of combat sports. These negative impacts during training may thus result in short-term training cessation and subsequently perturb athletic performance and competition preparations (Eston et al., 2003).

On the other hand, during intensified competitions or intense training, exercise-induced muscle damage has been associated with decreases in muscle strength, impaired muscle contractile functions, and impaired muscle recovery capacity (Knitter et al., 2000; Kendall and Eston, 2002), which may further impair subsequent competition performance. In addition, exercise-induced stress also markedly increases systematic inflammatory responses (Chou et al., 2018), cell damage (Chou et al., 2018), and catabolic/anabolic hormonal balance (Brownlee et al., 2005; Pilz-Burstein et al., 2010) in both general populations and combat sport athletes. Hence, it would be critical to develop practical training models with less physical contact while still being capable of mimicking the physiological reality of international competition and minimizing injury risks. It has also been shown that TKD combat simulation is not able to perfectly mimic TKD competition. During combat simulation, TKD athletes displayed similar exchange time, shorter preparation time, and a longer exchange preparation ratio compared to TKD competition using time-motion analysis (Hausen et al., 2017). Nevertheless, it is still little known whether competition-simulated exercise with less direct physical contact could completely reproduce the physiological responses of competition in addition to bring the benefit of lower injury risk.

To date, there is still very limited number of studies comparing the differences between simulated-kicking/fight-training and TKD competition in elite TKD athletes (Hausen et al., 2017; Maloney et al., 2018). One recent psychological study reported that the emotional and cognitive demands of competition cannot be simulated by fighting in training (Maloney et al., 2018), and Tayech et al. suggest that the anaerobic intermittent kick test can be used as a reliable and specific test for evaluating anaerobic power in TKD athletes (Tayech et al., 2019). Thus, it is still unclear whether a TKD-specific simulated training mode would be capable of replicating the physiological challenges of combat during intensive competitions. Moreover, to our knowledge, there is no study that has developed a low-impact fighting simulation training model and examined the relevant physiological variables in accordance with the Olympic competition time frame.

In TKD, kick technics are one of the primary attacking strategies (Pieter and Heijmans, 1997; Whang et al., 1999) and are very frequently used in competitions (Matsushigue et al., 2009; Kwok, 2012). According to the literature, for both medalists and nonmedalists, the main attack actions used during TKD combat are kicks (approximately 98% of overall attacks per match), such as roundhouse kick (63–69%), sidekick (6–7%), or reverse sidekick (1–2%) (Kwok, 2012; Bridge et al., 2014). Several studies have pointed out that the work-to-rest ratio (WRR) varied from 1:1 to 1:9 due to the level of games (Matsushigue et al., 2009; Santos et al., 2011; Campos et al., 2012; Del Vecchio et al., 2016). These varied WRRs across studies also create difficulties to reconstruct an appropriate simulation model for this combat sport. Although Matsushigue et al. (2009) reported a 1:1 WRR during a TKD match with 2-min rounds, the authors used Songahm Taekwondo competitions, which have very different rules compared to World Taekwondo. On the other hand, it has to be noted that the TKD athletes still have to perform high-intensity avoiding/dodging or other defending movements when they are not attacking, which may lead to an underestimation of real working time during combat. Considering these aspects, we reconstructed a competition-simulated kick model (TMSK) consisting of the most common kick techniques with a WTT of 1:1 (continuous 10-s kicking attacks) in order to elicit sufficient physiological stress and exercise intensity during the simulation. Consequently, we here developed a TKD-specific 2-min intermittent exercise model consisting of continuous 10-s shifting kicks (sidekick/reverse side kick/roundhouse kick)/10-s low intensity bouncing (1:1 WRR), attempting to reconstruct the physiological challenges during TKD combat, and compared this simulated intermittent kick model with a real TKD combat match. In this regard, we hypothesized that the TKD match-simulated kicking (TMSK) model would elicit comparable physiological stress and hormonal responses to the extent of TKD competition with an Olympic time frame. Therefore, the purpose of this study is to compare the effects of high-intensity intermittent match-simulated kicking and real TKD matches on the cardiopulmonary demands, physiological challenges, muscle-damaging biomarkers, and anabolic/catabolic hormonal responses in elite combat TKD athletes.

## Materials and Methods

### Participants

Sixteen elite TKD athletes (mean ± S.E.M.; age: 22.0 ± 0.3 years, height: 177.0 ± 1.8 cm, body weight: 71.0 ± 2.2 kg, BMI: 23.0 ± 0.4) participated in this study, and they were weight-matched and randomly assigned into either TMSK (*n* = 8) or STC (*n* = 8). All participants hold black belts, classifying in the National Division I category. The weight class of participants was classified based on Olympic weight class rules as follows: ≤58 kg (*n* = 2), >58 to ≤68 kg (*n* = 4), >68 to ≤80 kg (*n* = 6), >80 kg (*n* = 4). All combat matches were conducted by matching players with opponents at the similar skill levels in the same weight categories. Participants were free of musculoskeletal injuries and cardiovascular/metabolic disorders, determined by screening with a self-screening health questionnaire. This study was carried out in accordance with the recommendations of the Institute Review Board (IRB) of the University of Taipei with written informed consent from all subjects. All subjects gave written informed consent according to the Declaration of Helsinki.

### Experimental Design

A randomized and weight-matched study was used. During the trial day, all participants had the same meals. Before the first match, the participants consumed a breakfast provided by the researchers (energy: ~530 kcal; carbohydrate: 62%; fat: 20%; protein: 18%). Standardized snacks (energy: ~400–500 kcal; carbohydrate: 61–83%; fat: 5–20%; protein: 9–18%) were given immediately after each match, and dinner (energy: ~1,100 kcal; carbohydrate: 57%; fat: 25%; protein: 18%) was given after the last match (Match #4). The participants were requested to consume the provided food within 30 min to ensure consistency.

### Match-Simulated Kicking Mode and Simulated Competition

**Figure 1:**
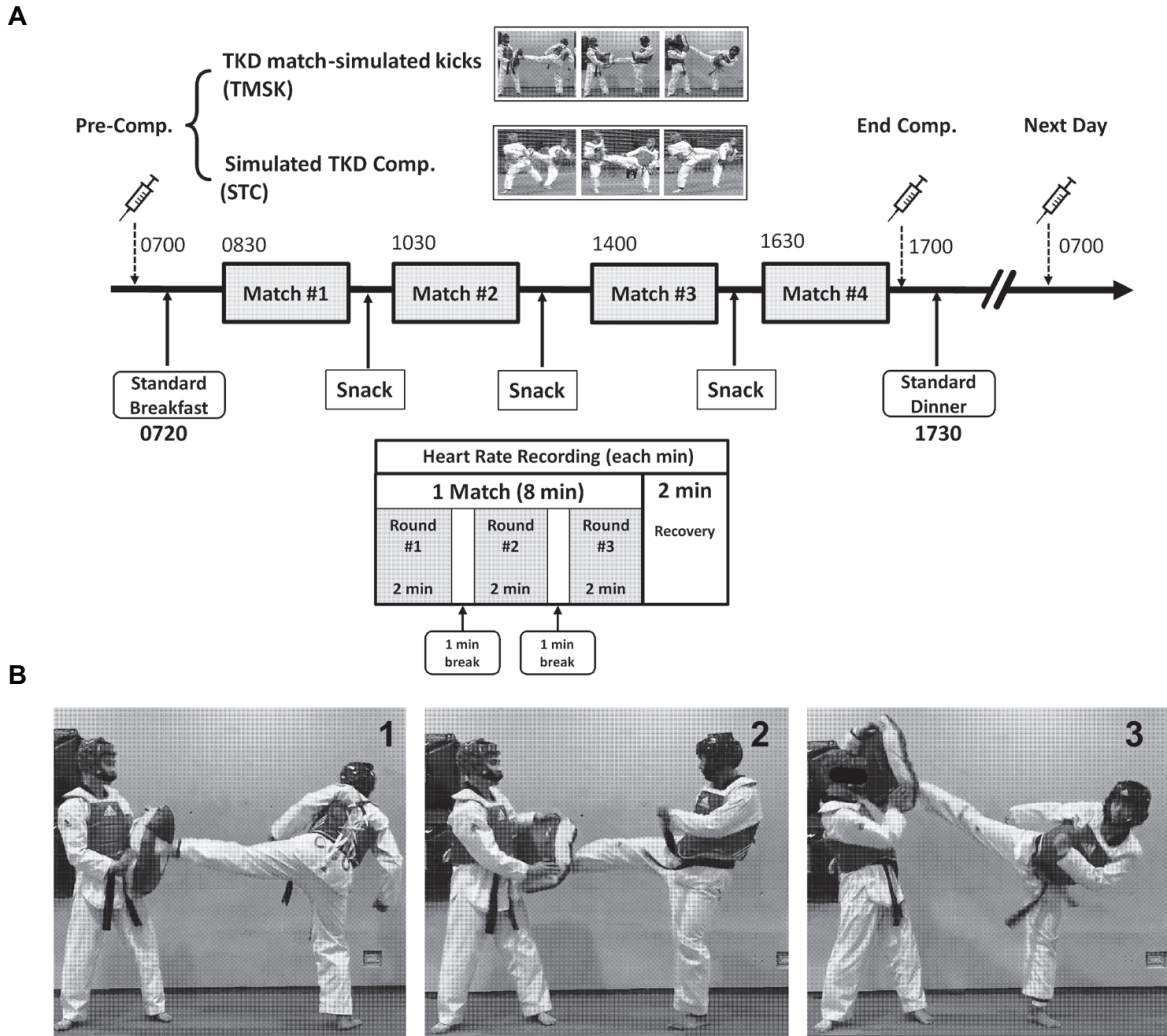
Experiment design and procedure of match in accordance with current Olympic TKD competition time frame. **(A)** Four consecutive combat matches were performed at 8:30, 10:30, 14:00, and 16:30. The blood samples were drawn before matches (7:00), after the completion of the last match (17:00), and on Next day (07:00; 24 h after the first match). **(B)** Three different kicks were used in the intermittent interval exercise (10-s kicking with full effort and 10-s bouncing jumping break) in the present study, and the movements used to reconstruct real TKD competition during the TMSK, including (1) sidekick, (2) reverse side kick, and (3) roundhouse kick. The individuals in this manuscript gave written informed consent to use and publish these image details. TMSK: TKD-match simulated kicks; STC: simulated TKD competition.

Participants in STC fought against an opponent in their matched weight category, which was based on Olympic weight regulation and equal experience to ensure the intensity of competition. The competition was performed in an 8 m × 8 m rubber mat octagonal competition area according to WT regulations and rules. Each match was composed of three rounds of 2 min and two breaks of 1 min. The consecutive competition (STC)/simulated matches (TMSK) were performed at 8:30 h (Match #1), 10:30 h (Match #2), 14:00 h (Match #3), and 16:30 h (Match #4) (Figure 1A). Participants wore the whole set of electronic protectors (2.7 kg; Dae do International, Barcelona, Spain) during simulated competition. For the TMSK, the athletes wore the whole set of regular protectors (2.1 kg; Adidas Double-d Martial Arts, Émerainville, France) during simulation, and the extra weight vest (0.6 kg) was used to balance the difference between the electronic and normal protectors. To simulate TKD match, subjects continuously kicked a soft target (Figure 1B) for 10 s as many times as possible and rested actively by low intensity bouncing for 10 s (1:1 WRR) between each trial of kicking to simulate a high-intensity interval pattern such as what would occur in competition. As sidekick/reverse sidekick/roundhouse kick are the primary choice of attack and accounted for 72–76% of the total attacks (Kwok, 2012; Bridge et al., 2014), subjects performed three different kicking technics in sequence in a simulation based on the time frame of an Olympic competition schedule. The heart rate was measured using a wireless heart rate monitor (Polar^®^ RS800CX™; Polar Electro Inc., Lake Success, NY, USA) during exercise and rest for both STC and TSMK groups.

### Measurement of Exercise Intensity and Fatigue

To monitor heart rate, a wireless heart rate monitor was attached to participants and the heart rate (HR) was periodically recorded during the experiment procedure. Blood lactate was measured from fingertip blood samples immediately post-match using a lactate meter (Edge Blood Lactate Monitoring System, ApexBio Inc., Taipei City, Taiwan). Borg rating of perceived exertion (RPE, 6–20 scale) was used to monitor exercise intensity, and the participants were familiarized with subjective scale prior to trial. During the STC or TMSK, the RPE rating was recorded at baseline, Round 1 (R1), Round 2 (R2), and Round 3 (R3) of each match.

### Analyses of Muscle Damage Biomarkers, Hematological Profiles, and Hormonal Levels

The 10-ml venous blood samples were collected in a tube coated with EDTA before Match #1 (Pre-Comp.), after completion of Match #4 (End-Comp.) and at 24 h after the first match (Next day). An automated hematology analyzer (Sysmex XT-2000, Sysmex Corp., Kobe, Japan) was used to analyze hematological profiles, including neutrophils, white blood cells (WBC), red blood cells (RBC), lymphocytes, and platelets, according to the manufacturer’s instructions. Both the neutrophils-to-lymphocytes ratio (NLR) and the platelet-to-lymphocytes ratio (PLR) have been clinically recognized as systemic inflammatory markers (Turkmen et al., 2013; Balta et al., 2016). Creatine kinase (CK) and myoglobin are commonly used indicators of muscle damage (Balnave and Thompson, 1993; Serrao et al., 2003). The concentration of plasma CK was determined using an LX-20 clinical chemistry analyzer (intra-assay *CV* = 7.5%; Beckman, Brea, CA, USA). Serum myoglobin was determined using radioimmunoassay by test kit (intra-assay *CV* = 1.5%; Daiichi Radioisotope Laboratory Ltd, Tokyo Japan). The circulating testosterone and cortisol levels were measured using commercially available enzyme-linked immunosorbent assay (ELISA) kits (testosterone: intra-assay *CV*% = 11.95%; #582701; cortisol: intra-assay *CV*% = 8.35%; #500360; Cayman Chemical Co., Ann Arbor, MI, USA), and the optical density of ELISA analyses was determined using a TECAN Genios reader (Salzburg, Austria) according to the manufacturer’s instructions.

### Statistical Analysis

All data were expressed as mean ± standard error of mean (Mean ± S.E.M.). Data were analyzed and depicted by SPSS 16.0 software (SPSS, Chicago, IL, USA) and GraphPad Prism 5.0 (GraphPad Software Inc., La Jolla, CA, USA), respectively. Prior to performing statistical analysis, all data were examined for normality of distribution. The heart rate, blood lactate, and blood biomarkers (e.g., creatine kinase, myoglobin, hematological profiles, hormonal responses, etc.) were analyzed using two-way mixed analysis of variance (2-way ANOVA; treatment group × time) with repeated measures to determine the intra- and inter-group differences. The effect size was calculated by Cohen’s f. When significance was found, *post hoc* testing (Tukey’s multiple comparisons test) was used to analyze the difference. The alpha level was set at 0.05 (*p* ≤ 0.05) for statistical difference.

## Results

### Physiology Response Measuring Heart Rate, RPE, and Lactate Levels

**Figure 2:**
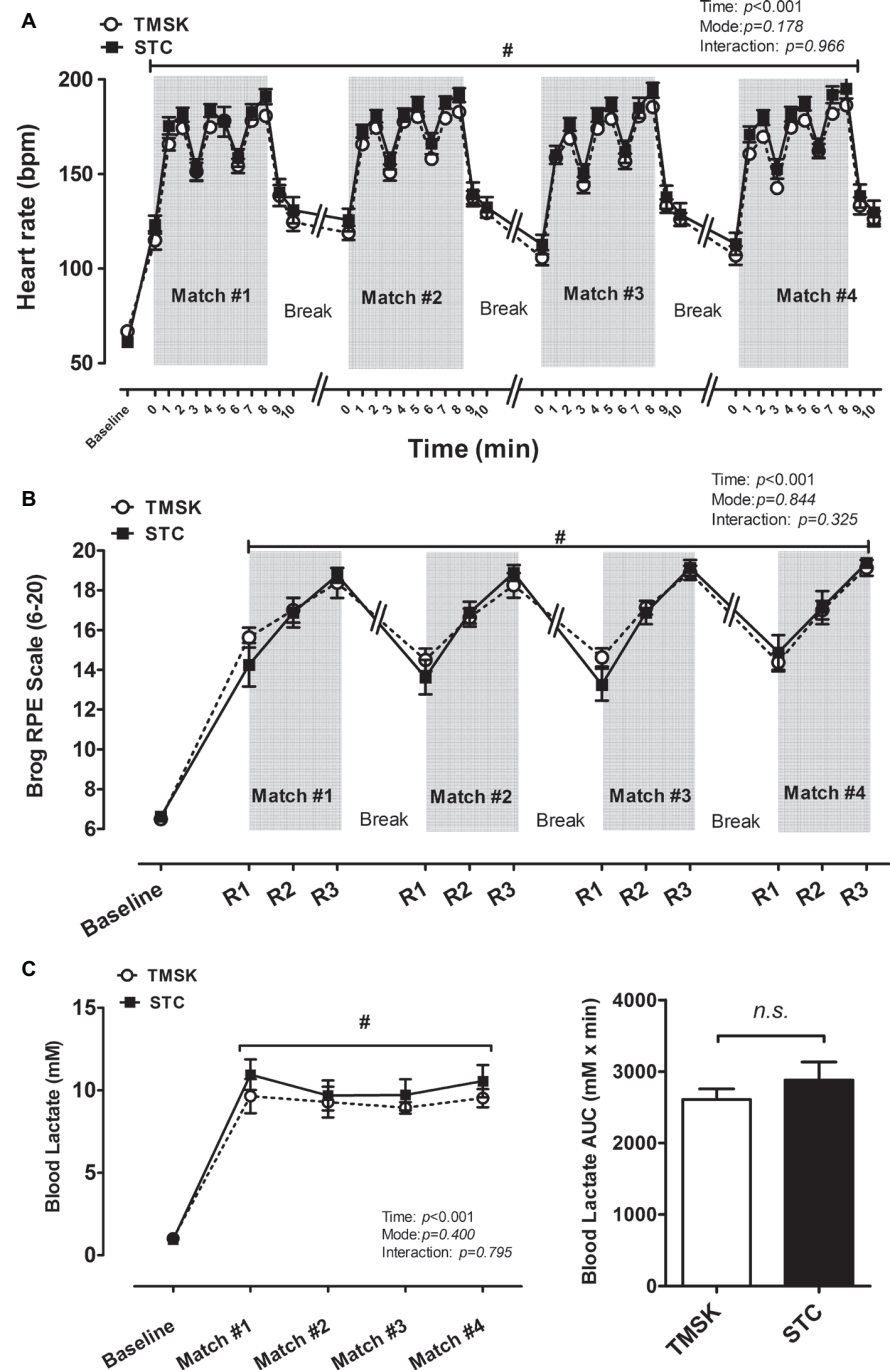
The physiological responses during real combat match and simulation kicking exercise. **(A)** heart rate, **(B)** blood lactate, and **(C)** subjective Borg RPE. # denotes significant difference compared to baseline values (*p* < 0.05). TMSK: TKD-match simulated kicks; STC: simulated TKD competition.

Figure 2 shows the changes in heart rate (Figure 2A), blood lactate (Figure 2B), and RPE (Figure 2C) during four TKD matches and the simulation. For heart rate, there was a significant main effect of time (*F*(44, 616) = 172.1, *p* < 0.001, *η*^2^ = 0.924) but not mode and interaction (Figure 2A). There was no effect of mode and interaction observed in RPE. Consistent with heart rate, RPE increased with time (*F*(12, 168) = 114.8, *p* < 0.001, *η*^2^ = 0.901) (Figure 2B). For lactate, as shown before in RPE, there was a significant main effect of time (*F*(4, 56) = 90.42, *p* < 0.001, *η*^2^ = 0.849) but not of mode or interaction (Figure 2C).

### Muscle Damage and Intravascular Hemolysis

**Figure 3:**
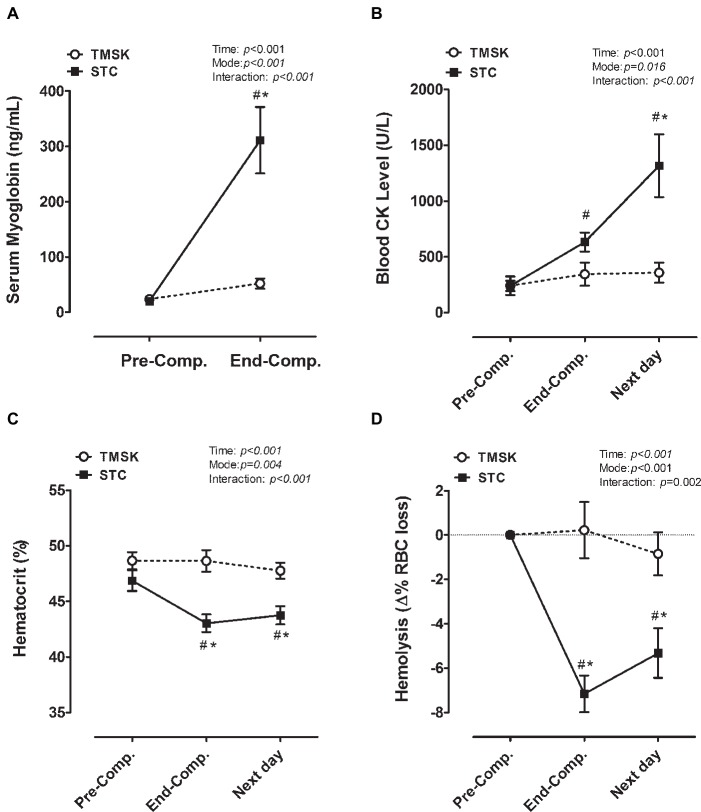
The responses of muscle-damaging blood biomarkers and status of hemolysis during real combat match and simulation kicking exercise. **(A)** Serum myoglobin, **(B)** blood CK, **(C)** hematocrit, **(D)** hemolysis. # denotes the significant difference compared to baseline values (*p* < 0.05); * denotes a significant difference between TMSK and STC (*p* < 0.05). TMSK: TKD-match simulated kicks; STC: simulated TKD competition.

Figure 3 shows the effect of TKD combat matches and the kicking simulation on muscle damage (Figures 3A,B) and intravascular hemolysis (Figures 3C,D). For serum myoglobin and CK levels, there was a significant main effect of time (myoglobin: *F*(1, 14) = 28.31, *p* < 0.001, *η*^2^ = 0.657; CK: *F*(2, 28) = 15.59, *p* < 0.001, *η*^2^ = 0.429), mode (myoglobin: *F*(1, 14) = 17.14, *p* < 0.001, *η*^2^ = 0.550; CK: *F*(1, 14) = 7.568, *p* = 0.016, *η*^2^ = 0.351), and interaction (myoglobin: *F*(1, 14) = 19.19, *p* < 0.001, *η*^2^ = 0.565; CK: *F*(2, 28) = 10.50, *p* < 0.001, *η*^2^ = 0.336). *Post hoc* analyses showed significant increases in myoglobin in STC at End-Comp (95% CI: 157.2–361.0, *p* < 0.001) and significant increases in CK level in STC at Next day (95% CI: 471.8–1,447, *p* < 0.001). Intravascular hemolysis can be measured by the decrease in red blood cells. For hematocrit and hemolysis, there was a significant main effect of time (hematocrit: *F*(2, 28) = 19.20, *p* < 0.001, *η*^2^ = 0.578; hemolysis: *F*(2, 28) = 14.58, *p* < 0.001, *η*^2^ = 0.510), mode (hematocrit: *F*(1, 14) = 11.69, *p* = 0.004, *η*^2^ = 0.852; hemolysis: *F*(1, 14) = 18.73, *p* < 0.001, *η*^2^ = 0.628), and interaction (hematocrit: *F*(2, 28) = 13.60, *p* < 0.001, *η*^2^ = 0.493; hemolysis: *F*(2, 28) = 13.95, *p* = 0.002, *η*^2^ = 0.499). *Post hoc* analyses showed significant decreasing in STC in hematocrit (End-Comp: 95% CI: −8.584 to −2.641, *p* < 0.001; Next day: 95% CI: −6.984 to −1.041, *p* = 0.005) and in hemolysis (End-Comp: 95% CI: −10.44 to −4.337, *p* < 0.001; Next day: 95% CI: −7.525 to −1.425, *p* = 0.002).

### Hematological Profiles and Inflammatory Parameters

**Figure 4:**
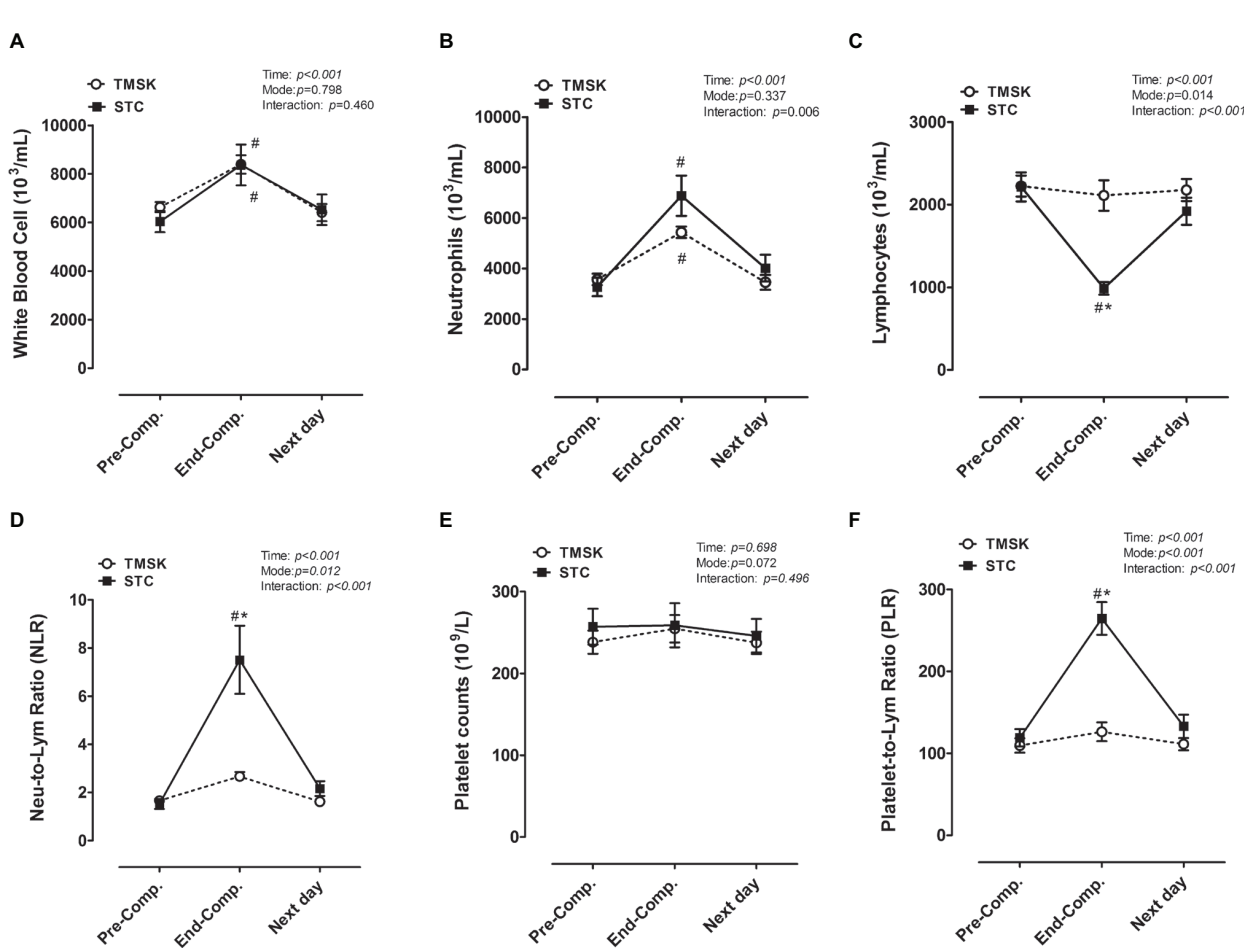
The responses of hematological profiles **(A-C,E)** and systemic inflammatory biomarkers **(D,F)** during real combat match and simulation kicking exercise. # denotes a significant difference compared to baseline values (*p* < 0.05); * denotes significant difference between TMSK and STC (*p* < 0.05). TMSK: TKD-match simulated kicks; STC: simulated TKD competition.

Figure 4 represents the changes of WBC (Figure 4A), neutrophils (Figure 4B), lymphocyte (Figure 4C), NLR ratio (Figure 4D), platelet (Figure 4E), and PLR ratio (Figure 4F) during matches or kicking simulation. For WBC, there was a significant main effect for time (*F*(2, 28) = 29.89, *p <* 0.001, *η*^2^ = 0.681) but not mode or interaction (Figure 4A), and both STC and TMSK increased WBC (TMSK: 95% CI: −2,740 to −787.9, *p* < 0.001; STC: 95% CI: −3,308 to −1,357, *p* < 0.001) at End-Comp., and the values returned to baseline at Next day. There was a significant main effect for time (*F*(2, 28) = 72.48, *p* < 0.001, *η*^2^ = 0.838) and interaction (*F*(2, 28) = 6.100, *p =* 0.006, *η*^2^ = 0.303) but not mode in neutrophils, and both STC and TMSK increased neutrophils (TMSK: 95% CI: −2,742 to −999.3, *p* < 0.001; STC: 95% CI: −4,481 to −2,739, *p* < 0.001) at End-Comp., and the values returned to baseline at Next day (Figure 4B). For lymphocytes, significant main effects for time (*F*(2, 28) = 19.03, *p* < 0.001, *η*^2^ = 0.576), mode (*F*(1, 14) = 7.959, *p* = 0.014, *η*^2^ = 0.474), and interaction (*F*(2, 28) = 13.39, *p* < 0.001, *η*^2^ = 0.489) were observed. Only STC significantly decreased lymphocytes at End-Comp. (95% CI: 855.9–1,600, *p* < 0.001) below the level of Pre-Comp., and the lymphocytes was significantly lower in STC than TMSK at End-Comp (95% CI: −1,647 to −600.5, *p* < 0.001) (Figure 4C). For the NLR ratio, a significant main effect for time (*F*(2, 28) = 26.79, *p* < 0.001, *η*^2^ = 0.657), mode (*F*(1, 14) = 8.271, *p* = 0.012, *η*^2^ = 0.369), and interaction (*F*(2, 28) = 12.99, *p* < 0.001, *η*^2^ = 0.481) was observed. Only STC significantly increased NLR at End-Comp. (95% CI: −182.6 to −108.6, *p* < 0.001) above the level of Pre-Comp, and the NLR was significantly higher in STC than TMSK at End-Comp (95% CI: 93.34–183.4, *p* < 0.001) (Figure 4D). The platelet counts did not show any significant differences during TKD matches, and there were no differences between the two groups (Figure 4E). For the PLR ratio, a significant main effect of time (*F*(2, 28) = 31.72, *p* < 0.001, *η*^2^ = 0.694), mode (*F*(1, 14) = 20.23, *p* < 0.001, *η*^2^ = 0.575), and interaction (*F*(2, 28) = 20.06, *p* < 0.001, *η*^2^ = 0.589) was observed. Only STC significantly increased PLR at End-Comp. (95% CI: −182.6 to −108.6, *p* < 0.001) above the level of Pre-Comp, and the PLR was significantly higher in STC than TMSK at End-Comp (95% CI: 93.34–183.4, *p* < 0.001) (Figure 4F).

### Anabolic/Catabolic Hormone Responses to Taekwondo Matches

**Figure 5:**
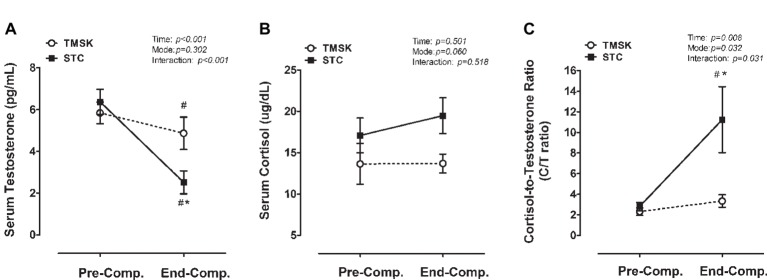
The responses of anabolic and catabolic hormones and balance during real combat match and simulation kicking exercise. **(A)** Serum testosterone, **(B)** serum cortisol, and **(C)** C/T ratio. # denotes the significant difference compared to baseline values (*p* < 0.05); * denotes significant difference between TMSK and STC (*p* < 0.05). TMSK: TKD-match simulated kicks; STC: simulated TKD competition.

Figure 5 indicates the changes of circulating testosterone (Figure 5A), cortisol (Figure 5B), and systemic catabolic index presented by C/T ratio (Figure 5C) during simulated competition or kicking simulation. For serum testosterone, a significant main effect for time (*F*(1, 14) = 127.3, *p* < 0.001, *η*^2^ = 0.901) and interaction (*F*(1, 14) = 45.31, *p* < 0.001, *η*^2^ = 0.764), but no effect for mode was observed. *Post hoc* analyses revealed that TMSK (95% CI: −1.735 to −0.2151, *p* = 0.012) and STC (95% CI: −4.617 to −3.098, *p* < 0.001) significantly decreased serum testosterone during competition/simulation, and TMSK further significantly reduced at end-comp (95% CI: −4.423 to −0.2792, *p* = 0.024) (Figure 5A). After competition/simulation, there was no effect of time, mode, or interaction in serum cortisol (Figure 5B). For the C/T ratio, there was an effect for time (*F*(1, 14) = 9.417, *p* = 0.006, *η*^2^ = 0.402), mode (*F*(1, 14) = 5.701, *p* = 0.032, *η*^2^ = 0.347), and interaction (*F*(1, 14) = 5.770, *p* = 0.031, *η*^2^ = 0.29). *Post hoc* analyses indicated that STC significantly increased C/T ratio (95% CI: 2.958–13.88, *p* = 0.003), and the C/T ratio was significantly higher in STC than TMSK at End-comp. (95% CI: 2.362–13.44, *p* = 0.004) (Figure 5C).

## Discussion

The aim of the present research was to compare the effects of TMSK and TKD competition matches on physiological challenges, exercise-induced muscle damage, and hormonal responses in elite combat TKD athletes. To our knowledge, this study is the first to compare the effect of kicking simulation and simulated competition using an Olympic competition time frame on physiological challenges. The primary findings of this study were that (1) both TKD competition (STC) and kicking simulation (TMSK) were high-intensity intermittent exercise and elicited comparable challenges in heart rate, perceived exertion, and lactate responses; (2) compared with TMSK, the STC produced markedly greater muscle damage and inflammatory responses in the Olympic competition time frame; (3) furthermore, the STC provokes a relatively stronger systemic catabolic stress, reflected by an increase in the ratio of cortisol to testosterone after the consecutive combat matches, whereas there were no such response observed in TMSK group. Here, we demonstrated that the match-simulating kicking practice was capable of eliciting comparable physiological challenges as that of TKD competition, and no obvious exercise-induced muscle damage was observed. However, we found significant differences in systemic inflammatory and catabolic stresses between the combat and simulated kicking exercise models. Our present findings, therefore, provide a better understanding of the physiological diversities between match-based simulation training and combat competition for Olympic Taekwondo preparation.

### Physiological Characteristics of TMSK and STC

The data of physiological responses demonstrated that TMSK elicited a similar heart rate, perceived exertion rating, and lactate response compared to the STC (Figures 2A–C), indicating that both TMSK and STC can be recognized as high-intensity intermittent exercise (HIIE). Our results thus suggest that the match-simulated kicking exercise (TMSK) was capable of imitating and reconstructing physiological challenges and energy demands during TKD combat in an Olympic competition time frame. To our knowledge, this is the first study using a simulated kicking model combined with a schedule containing consecutive competitions (four simulations/matches in one day) alongside techniques for investigating relevant physiological responses according to Olympic TKD regulations. Previous studies regarding TKD training or competition are mostly based on technical/tactical training, physiological responses, or nutrient supplementations applying 1–4 simulation or combat matches (Bridge et al., 2009; Bridge et al., 2011; Casolino et al., 2012; Lopes-Silva et al., 2015; Chou et al., 2018), but only a few of these studies used the exact time frame of current Olympic competition regulations to investigate the changes in the relevant physiological biomarkers (Hausen et al., 2017). Some discrepant results from different studies might be explained by the amount of simulation/competition and different WRRs. Particularly, in the present study, we used here a longer period of simulation kicks with 1:1 WRR (attack time lasted for 10 s), which might result in higher accumulative physiological stress on athletes. In addition, the current Olympic TKD regulations have continued to be modified and updated (e.g., duration of combat round and between-round rest, contest area specifications, scoring regulations, etc.) (World Taekwondo, 2018), thus the investigations of physiological responses during combat using the most updated international rules is warranted for assisting in the preparation of a development plan for this sport.

In the Olympics, athletes must perform 4–5 consecutive competitions in 1 day to proceed to the final competition, and a classical match includes 3–4 rounds with 1-min intervals (i.e., three regular rounds plus one golden round if necessary) (World Taekwondo, 2018). Following the Olympic competition time frame, we herein observed that the heart rate, RPE, and blood lactate level were dramatically increased from Round 1 to Round 3 during each match in both STC and TMSK modes (maximal HR ranged from 181 to 195 bpm; maximal RPE ranged from 18.8 to 19.4; maximal lactate ranged from 8.94 to 10.95 mM), indicating that the physiological stresses also increased progressively across matches in both experimental modes. Our findings indicate that the TMSK-induced HR, PRE, and lactate responses are comparable to our present simulation combat mode (Figures 2A–C) and previous TKD studies using simulation combats (Campos et al., 2012; Lopes-Silva et al., 2015; Hausen et al., 2017). For example, the peak heart rate and peak blood lactate gradually increased from 181 to 189 bpm and from 8.4 to 12.3 mM after each round during a consecutive 3-round simulation TKD combat, respectively (Hausen et al., 2017). Furthermore, Lopes-Silva et al. (2015) also reported a gradually increasing peak heart rate and RPE from 177 to 188 bpm and from 14 to 18, respectively. These findings suggest that TMSK elicited adequate physiological challenges to the extent of simulated combat competitions. However, the peak heart rate (increasing from 175 to 187 bpm) and blood lactate (increasing from 7.5 to 11.9 bpm) responses appear to be slightly lower in competitions (Bridge et al., 2009) than those of simulated combat. One possible explanation for the differences between combat simulation and real competition might be the preparation time and exchange time/preparation time ratio (ET:PT ratio) (Bridge et al., 2009; Hausen et al., 2017). In combat simulation, athletes spend less time in preparation, and the ET:PT ratio is also lower, indicating that athletes may attack more in the simulation. In addition, the slightly higher physiological stress (heart rate, RPE, and blood late) may be contributed by the 4-match simulation of 1:1 WRR used in present study, as previous studies used from 1 to 3 matches with different WRRs. Consequently, in the present study, TMSK is capable of providing comparable similarities in intensity and physiological stress as STC in terms of international-level TKD combats.

### TKD Competition Induced More Tissue Damages Than Simulated Kicking Exercise

In competition, in addition to attacks, the TKD athletes themselves must also perform defensive skills and dodge, thus TKD athletes need to use some parts of the body to resist the opponent’s attack to prevent the opponent from scoring. Both attacking and resisting cause the body to hit a hard part or be hit by a hard part (joints, bones, etc.), thus causing more marked tissue damages. As a consequence, the nature of TKD combat may result in server muscle damage from the direct contacts during the matches (Bridge et al., 2009; Campos et al., 2012; Kwok, 2012). Singh et al. compared the effects of physical contact-induced muscle soreness on exercise performance during team sports, and they found that impact contact caused greater negative effects on subsequent performance with even stronger muscle discomfort/damage (Singh et al., 2011). In the present study, we observed that, compared to STC, the exercise-induced increasing myoglobin and CK were significantly lower in TMSK, suggesting that the simulated kicking did not provoke the same degree of muscle damage as TKD competition under comparable exercise intensity (Figures 3A,B). According to our present findings, the greater muscle damage in STC is probably due to direct physical contacts during attack and defense. Our observations are in line with the study by Takarada et al. that the blood muscle damage markers (myoglobin and CK) are closely associated with the number of tackles in a competitive rugby match (Takarada, 2003). Furthermore, mechanical trauma during exercise such as repeated physical impacts occurring at long-distance running is a major cause of intravascular hemolysis (Miller et al., 1988; Janakiraman et al., 2011). Although the frequency of direct physical contact during TKD combat may not be as high as long-distance running, each impact by defending or attacking during TKD combat can be much greater than each foot impact during long-distance running, which might lead to a substantial increase in intravascular hemolysis in STC group (Figure 3D). These results therefore suggest that, in the present study, the soft-target kicking simulation exercise yielded relatively lower tissue damages (i.e., muscle damage and hemolysis) compared with TKD matches, which might thereby prevent the increase in exercise-induced inflammation.

### Diverse Responses of Systemic Inflammation and Catabolic Stress Between TMSK and STC

Numerous studies have revealed that exhaustive exercise or eccentric exercise can lead to myofibrillar disruptions and initiate the inflammatory response (Tidball, 1995; Suzuki et al., 1999; Nosaka et al., 2002; Sayers and Clarkson, 2003). Neutrophils are rapidly infiltrated into damage tissue after exercise-induced muscle damage and evoke initial local inflammation (Kanda et al., 2013). Moreover, the NLR and PLR are widely applied to reflect the systemic inflammatory status in athletes and patients (Turkmen et al., 2013; Lou et al., 2015; Liao et al., 2016; Chen et al., 2017). In this study, we found the NLR and PLR were significantly higher in STC than in TMSK following the consecutive tasks, indicating that the STC provoked even greater systemic inflammatory responses (Figures 4D,F). In addition, we also observed that the STC but not TMSK significantly decreased the number of circulating lymphocytes (Figure 4C). This was in agreement with previous studies that the lymphocyte cell viability markedly decreased after exercise (Pedersen and Toft, 2000; Fisher et al., 2011). It has to be noted that only STC provoked a marked increase in muscle damage, hemolysis, and inflammatory responses after successive tests (Figures 3A,B), which is consistent with the finding by Singh et al. that only direct full contact produced a significant inflammatory response during exercise (Singh et al., 2011). As a result, these data support the postulation that the lymphocytes might be more susceptible to cytotoxic agents released from damaged muscle during post-exercise recovery, thereby changing the balance of immune cells’ profiles. However, the precise physiological mechanisms underlying the inflammatory responses to the consecutive full-contact taekwondo combat warrant further investigations.

On the other hand, the ratio between cortisol and testosterone (C/T ratio), the two primary catabolic and anabolic hormones, has been used to determine the degree of systemic catabolic stress in response to exercise (De Luccia, 2016). Here we observed that the C/T ratio dramatically increased following consecutive TKD competitions, but the C/T ratio was sustained at a relatively lower level in participants with simulated intermittent kicking exercise (Figure 5C). The present finding revealed clear accumulative systemic catabolic stress caused by the consecutive TKD competitions but not by the simulated kicking exercise. Corresponding to previous studies (Suzuki et al., 1999; Bishop et al., 2003; Davison and Gleeson, 2006), the circulating ratio between cortisol and testosterone markedly rose in response to strenuous exercise. Additionally, the circulating acute muscle damage biomarkers have been reported to be associated with catabolic endocrine profiles in team sport competitions (McLellan et al., 2010; Thorpe and Sunderland, 2012), therefore the elevated muscle damage appears to, at least in part, account for the STC-induced catabolic state after the consecutive TKD combats.

In this study, although our present findings revealed similar physiological and subjective responses (e.g., heart rate, lactate, and RPE) to the exercise loads between TMSK and STC modes, we are aware that other studies have done similar work and are based on closer WRRs (e.g., range of 1:3–1:5) of the observed ones in the modality to determine the influence on internal load and neuromuscular load responses in TKD athletes (Matsushigue et al., 2009; Santos et al., 2011; Campos et al., 2012; Del Vecchio et al., 2016; Sant’Ana et al., 2017). Additionally, the heart rate and blood lactate responses seem slightly lower during international competitions (Bridge et al., 2009). Hence, there still exists a certain degree of discrepancy to the external load actually observed during taekwondo competitions in this study, and this limitation might require further that studies apply even closer WRRs to investigate these relevant internal/external parameters using the Olympic time frame.

## Practical Perspectives

Taekwondo competition results in considerable physiological challenges plus tissue damage (i.e., muscle damage and hemolysis) through physical contact. Based on the finding that the TMSK mode yields less tissue damage, TKD coaches and conditioning specialists can incorporate TMSK into daily and regular training programs (e.g., strength training or drill/tactical/skill practice) to synergistically optimize the athlete’s physical condition and to help athletes to familiarize themselves with competition arrangement. Moreover, the marked increases in muscle damage and catabolic stress remained at high levels for at least 24 h after the competition, indicating that the coaches and sport scientists must take this into consideration for developing proper post-exercise recovery strategies (e.g., nutritional intervention, recovery modality, alternative approach, etc.) for these athletes. Our present data not only deliver the basic information about physical condition following competition but also provide a new conditioning training protocol complying with the Olympic competition time frame.

## Conclusion

The simulation protocol (TMSK) used in the present study elicited comparable physiological challenges to taekwondo competition but did not induce catabolic stress to the same extent as competition during the consecutive competitions under an Olympic time frame in elite TKD athletes. Although TMSK produced similar heart rate and lactate as real TKD competition, the muscle damage, inflammatory responses, and catabolic stress brought about by the simulated kicking exercise were relatively lower compared to the real TKD competition. The fundamental information provided by the present study may help coaches, athletes, and sport scientists to develop a more realistic TKD-specific training program and post-competition/training recovery strategy for Olympic preparation.

## Author Contributions

C-CC, Y-HL, and Y-CS conceived and designed the experiments. Y-HL, C-YC, and Y-CS performed the experiments. Y-HL, S-JC, and C-CC analyzed the data. S-JC, Y-CS, Y-HL, and C-YC wrote the paper. Y-HL, C-CC, and Y-CS contributed reagents, materials and analysis tools.

### Conflict of Interest Statement

The authors declare that the research was conducted in the absence of any commercial or financial relationships that could be construed as a potential conflict of interest.
